# Assessing the repeatability of expiratory flow limitation during incremental exercise in healthy adults

**DOI:** 10.14814/phy2.70068

**Published:** 2024-10-02

**Authors:** Jack R. Dunsford, Jasvir K. Dhaliwal, Gracie O. Grift, Robert Pryce, Paolo B. Dominelli, Yannick Molgat‐Seon

**Affiliations:** ^1^ Department of Kinesiology and Applied Health University of Winnipeg Winnipeg Manitoba Canada; ^2^ Department of Kinesiology and Health Sciences University of Waterloo Waterloo Ontario Canada; ^3^ Centre for Heart and Lung Innovation St. Paul's Hospital Vancouver British Columbia Canada

**Keywords:** expiratory flow limitation, repeatability, ventilatory capacity, ventilatory constraint, ventilatory demand

## Abstract

We sought to determine the repeatability of EFL in healthy adults during incremental cycle exercise. We hypothesized that the repeatability of EFL would be “strong” when assessed as a binary variable (i.e., absent or present) but “poor” when assessed as a continuous variable (i.e., % tidal volume overlap). Thirty‐two healthy adults performed spirometry and an incremental cycle exercise test to exhaustion on two occasions. Standard cardiorespiratory variables were measured at rest and throughout exercise, and EFL was assessed by overlaying tidal expiratory flow‐volume and maximal expiratory flow‐volume curves. The repeatability of EFL was determined using Cohen's *κ* for binary assessments of EFL and intraclass correlation (ICC) for continuous measures of EFL. During exercise, *n* = 12 participants (38%) experienced EFL. At peak exercise, the repeatability of EFL was “minimal” (*κ* = 0.337, *p* = 0.145) when assessed as a binary variable and “poor” when measured as a continuous variable (ICC = 0.338, *p* = 0.025). At matched levels of minute ventilation during high‐intensity exercise (i.e., >75% of peak oxygen uptake), the repeatability of EFL was “weak” when measured as a binary variable (*κ* = 0.474, *p* = 0.001) and “moderate” when measured as a continuous variable (ICC = 0.603, *p* < 0.001). Our results highlight the day‐to‐day variability associated with assessing EFL during exercise in healthy adults.

## 1 INTRODUCTION

In humans, the ventilatory demand–capacity relationship of the respiratory system describes the extent to which minute ventilation (V̇_E_) encroaches on the system's finite ability to generate airflow (Dempsey et al., 2020; Johnson & Dempsey, 1991). Ventilatory demand, which reflects whole‐body metabolism, is determined based on tidal volume and tidal expiratory flow, while ventilatory capacity is represented by maximal expiratory flow across vital capacity. When V̇_E_ is relatively low, as is the case at rest or during low‐ to moderate‐intensity exercise, there is substantial inspiratory and expiratory reserve, whereby tidal expiratory flow is well below volume‐specific maximal expiratory flow, indicating that ventilatory capacity exceeds ventilatory demand (Peters et al., 2023). However, in some cases (e.g., during high‐intensity exercise), ventilatory demand can approach, meet, or even exceed ventilatory capacity resulting in expiratory flow limitation (EFL) (Peters et al., 2023). This phenomenon occurs when tidal expiratory flow reaches maximal expiratory flow and cannot be further increased even with increased muscle effort (Hyatt, 1983). In healthy adults, EFL occurs at peak exercise in ~50% of individuals (Molgat‐Seon et al., 2022), and has been shown to increase respiratory muscle work, decrease exercise tolerance, increase the perception of dyspnea, as well as negatively impact blood gas homeostasis, blood volume distribution, and the regulation of operating lung volumes (Dominelli et al., 2013, 2014; Iandelli et al., 2002; Pellegrino et al., 1993; Stucky et al., 2023).

**FIGURE 1:**
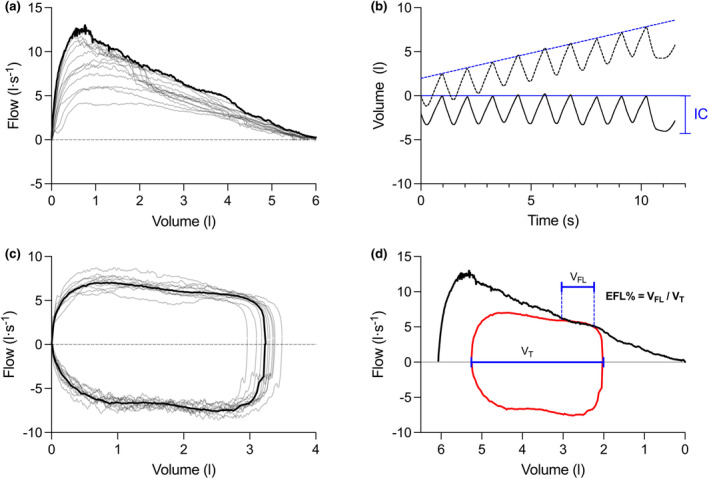
Graphical representation of the analyses involved in assessing EFL. In (a) the MEFV curve is generated by taking the highest flow for every lung volume across all graded FVC maneuvers performed before and after exercise. In (b) the volume signal is drift‐corrected using a linear regression of end‐expiratory volumes. The IC is then calculated as the lowest volume relative to the average end‐expiratory lung volume. In (c) after correcting for drift in the volume signal, ~30 s of flow and volume data for each stage are separated into individual breaths and an average tidal flow‐volume curve is generated. In (d) the presence of EFL is evaluated based on the presence or absence of overlap between the tidal expiratory flow‐volume curve and the MEFV curve. If EFL is present, the magnitude of EFL (%EFL) is calculated by determining the fraction of tidal volume that is flow‐limited (V_FL_). EFL, expiratory flow limitation; IC, inspiratory capacity; V_FL_, fraction of tidal volume that is flow‐limited; V_T_, tidal volume.

Although there are several methods of assessing EFL (Tantucci, 2013), the most common involves overlaying a tidal expiratory flow‐volume curve and the maximum expiratory flow‐volume (MEFV) curve based on operating lung volumes (Bartlett Jr. et al., 1963; Grimby et al., 1971; Hyatt, 1961). When a portion of tidal expiratory flow‐volume curve overlaps with the MEFV curve, EFL is considered present (Bartlett Jr. et al., 1963; Grimby et al., 1971; Hyatt, 1961; Stickland et al., 2022) (Figure 1d). Using this method, EFL can be assessed as a binary variable (i.e., present or absent) or as a continuous variable (i.e., % of expiratory tidal volume overlapping the MEFV curve) but requires several key measures (Figure 1a–c). First, an average tidal expiratory flow‐volume curve must be generated for a given time period of interest (Dominelli & Sheel, 2012). Second, the inspiratory capacity (IC) that corresponds to the same time period of interest must be measured to calculate operating lung volumes (Guenette et al., 2013). Finally, an MEFV curve must be generated by accounting for the effects of thoracic gas compression and changes in bronchial tone (Guenette et al., 2010). Not only are these measures technically demanding, but they require practice and compliance from the participant, and any degree of error in each of the aforementioned measures directly influences the assessment of EFL.

Despite the widespread use of flow‐volume curve analysis for the assessment of EFL (Koulouris & Hardavella, 2011), the repeatability of the EFL in healthy adults using this technique has not been established. The repeatability of EFL using the negative expiratory pressure technique has been examined in patients with chronic obstructive pulmonary disease (COPD) at rest, and it appears that EFL is repeatable when assessed as a binary variable but not as a continuous variable (Chen et al., 2010; Walker et al., 2007). Although patients with COPD are more likely to experience EFL than healthy adults (Smith et al., 2017) and that there are important methodological differences between the two methods of assessing EFL, it is reasonable to surmise that the repeatability of EFL during exercise in healthy adults assessed by overlaying tidal expiratory flow‐volume and MEFV curves would follow a similar pattern. Establishing the repeatability of EFL during exercise in healthy adults using this method has important implications for studies with repeated measures of EFL.

The purpose of this study was to determine the repeatability of EFL during exercise in healthy adults by analyzing tidal expiratory flow‐volume and MEFV curves. Our hypotheses were twofold: (i) when treated as a binary measure, the repeatability of EFL during exercise would be “strong,” and (ii) when assessed as a continuous measure, the repeatability of EFL during exercise would be “poor.”

## 2 METHODS

### 2.1 Research design and participants

Thirty‐two (*n* = 20 males, *n* = 12 females) participants completed the study. All participants provided written informed consent and all study procedures were approved by the University of Winnipeg's Human Research Ethics Board (#HE17119), which adheres to the *Declaration of Helsinki*, except for registration in a database. Participants were included if they: were between the ages of 20 and 60 years, inclusively, had a body mass index ≥18 and ≤30 kg·m^−2^, and had normal pulmonary function based on predicted normal values (Quanjer et al., 2012). Participants were excluded if they had a history or current symptoms of cardiovascular or pulmonary disease, a previous history of smoking, or any contraindications to cardiopulmonary exercise testing.

### 2.2 Experimental overview

Participants reported to the laboratory on two separate occasions separated by a minimum of 48 h. During each visit, participants underwent basic anthropometric and pulmonary function measurements followed by 5–10 min of rest before completing an incremental cycle exercise test to exhaustion. Before and after exercise, participants performed a series of forced vital capacity (FVC) maneuvers to construct a MEFV curve (see Maximum Expiratory Flow‐Volume Curve below).

### 2.3 Exercise protocol

During each visit, participants performed an incremental exercise test on an upright electronically braked cycle ergometer (Ergoselect 200 K, Ergoline GmbH, Bitz, Germany) according to standard recommendations (American Thoracic Society, and American College of Chest Physicians, 2003). The test was preceded by 2 min of steady‐state rest and began at a workload of 20 W followed by a stepwise increase in work rate by 20 W every 2 min until volitional exhaustion. A maximal effort was confirmed based on standard criteria (American Thoracic Society, and American College of Chest Physicians, 2003).

### 2.4 Measurements

#### 2.4.1 Pulmonary function

Participants completed spirometry according to standard recommendations (Graham et al., 2019) using a portable spirometer (Mini‐Spir, Roxon, St‐Leonard, QC, Canada). All spirometry measures were expressed as absolute values and as a percentage of predicted normal values (Quanjer et al., 2012). Participants were also given thorough training and visual feedback on how to perform IC and graded FVC maneuvers prior to the start of the exercise test.

#### 2.4.2 Cardiorespiratory responses to exercise

At rest and throughout exercise, standard cardiorespiratory variables were measured using a customized system involving open‐circuit spirometry and expired gas analysis. Participants breathed through a low resistance circuit (i.e., 0.5–1.0 cm H_2_O l^−1^·s^−1^ at 0.5–8.1·s^−1^) that consisted of a mouthpiece, connected to a two‐way nonrebreathing valve (Model 2700, Hans Rudolph, Shawnee, KS, USA) with large‐bore tubing on the inspired and expired limbs. The inspired and expired tubing were connected to independent pneumotachometers (Models 3813 and 4813, Hans Rudolph, Shawnee, KS, USA) to measure airflow. Mixed expired oxygen and carbon dioxide fractions were sampled from a mixing chamber connected to the expiratory limb of the breathing circuit and measured using a gas analyzer (14–10,000, CWE Inc., Ardmore, PA, USA). Arterial oxygen saturation was estimated using finger pulse oximetry (Model 7500, Nonin Medical Inc., Plymouth, MN, USA). Heart rate was measured using a telemetric sensor (PTK25, AD Instruments, Colorado Springs, CO, USA). Standard cardiorespiratory variables, including oxygen uptake (V̇O_2_), carbon dioxide production, tidal volume, breathing frequency, V̇_E_, heart rate, and arterial oxygen saturation were averaged over 30s epochs at rest and throughout exercise.

#### 2.4.3 Maximum expiratory flow‐volume curve

Before and after each incremental exercise test, participants performed a series of FVC maneuvers at varying efforts from which a MEFV curve could be constructed by accounting for the effects of exercise‐induced changes in bronchial tone and thoracic gas compression (Guenette et al., 2010). To perform the FVC maneuvers, participants were instructed to take a deep breath from functional residual capacity to total lung capacity and immediately exhale rapidly and maximally to residual volume while seated on the cycle ergometer in the cycling position. Participants performed FVC maneuvers at varying expiratory efforts ranging from ~20% to 100% of maximum to account for the effect of thoracic gas compression on maximal expiratory flows. Each participant performed a minimum of eight acceptable FVC maneuvers to ensure that a wide range of expiratory efforts were achieved. The same procedure was repeated beginning within 1 min following the end of the incremental exercise test to account for the effect of exercise‐induced changes in bronchial tone on maximal expiratory flows. In total, each participant performed at least 16 FVC maneuvers, all of which were anchored to total lung capacity and the highest flow achieved was determined for any given absolute lung volume from total lung capacity to residual volume in 0.01 L increments (Figure 1a). Slope ratio (SR) provides a unitless measure used to describe the shape of the MEFV curve and was calculated by determining the ratio between the instantaneous tangent and chord slopes of any point along the MEFV curve, as previously described (Dominelli et al., 2016). We averaged SR across all points along the effort‐independent portion of the MEFV curve (20%–80% of FVC).

#### 2.4.4 Operating lung volumes

At rest and during the last 30 s of each exercise stage, participants performed an IC maneuver to determine operating lung volumes, as previously described (Guenette et al., 2013). Briefly, approximately 30 s of bidirectional flow and volume data, including the IC maneuver, were analyzed. Baseline end‐expiratory volume prior to the IC maneuver was established by excluding any aberrant breaths or anticipatory changes in breathing pattern and by accounting for drift in the volume signal. The IC was then determined by calculating the difference between the baseline end‐expiratory volume and the nadir volume achieved during the IC maneuver (Figure 1b). The IC was then subtracted from FVC to obtain expiratory reserve volume (ERV), and the corresponding tidal volume was added to ERV to obtain inspiratory reserve volume (IRV).

#### 2.4.5 Tidal flow‐volume curves

At rest and for each stage of exercise, 30 s of bidirectional flow and volume data prior to the IC maneuver were composite averaged to generate a tidal flow‐volume curve (Figure 1c). Theoretical maximum ventilation (V̇_E,CAP_) was calculated at rest and for each exercise stage based on the lung volume‐specific maximal expiratory flow from the MEFV curve and the corresponding tidal volume, as previously described (Johnson et al., 1999).

#### 2.4.6 Expiratory flow limitation

The presence of EFL was determined at rest and during each exercise stage by analyzing tidal expiratory flow‐volume and MEFV curves (Johnson et al., 1999) (Figure 1d). Briefly, tidal expiratory flow‐volume curves from rest and each stage of exercise were placed within the participant's MEFV curve based on the ERV derived from the corresponding IC maneuver. Once the average tidal flow‐volume curves were positioned within the MEFV curve, the presence of EFL was determined at rest and for each stage of exercise by assessing whether the expiratory limb of each average tidal flow‐volume curve overlapped with the MEFV curve. The fraction of the expiratory limb of the tidal flow‐volume curve that overlapped with the MEFV curve was calculated; EFL was said to present if the tidal expiratory flow‐volume curve encroached on the MEFV curve over ≥5% of tidal volume.

### 2.5 Data analysis

All data were sampled at 200 Hz using a 16‐channel analog‐to‐digital converter (PowerLab 16/35, AD Instruments, Colorado Springs, CO, USA) connected to a computer and recorded using commercially available software (LabChart Pro v8.1.17, AD Instruments, Colorado Springs, CO, USA). Respiratory data were analyzed using a custom Python script, which took manually selected segments of flow and volume data for each stage of exercise as well as FVC maneuvers from before and after exercise as input. For each participant, the program then calculated the MEFV curve, and for each segment of data (i.e., 30 s at rest and at the end of each exercise stage), averaged flow and volume for inspiration and expiration, calculated the IC while correcting for signal drift, calculated V̇_E,CAP_, and assessed EFL.

### 2.6 Statistical analysis

Participant characteristics as well as pulmonary function and peak exercise data were compared between visits 1 and 2 using Student's paired *t*‐test. The occurrence of EFL during exercise on visits 1 and 2 were expressed as frequency statistics and compared between visits using Fischer's exact test. A two‐way repeated measures analysis of variance was used to determine the effect of work rate and visit on the submaximal metabolic and ventilatory responses to incremental exercise. If significant *F* ratios were detected, pairwise comparisons were performed with Bonferroni corrections. Given that in healthy adults, EFL only occurs during high‐intensity exercise (Dominelli et al., 2011; Guenette et al., 2007; Molgat‐Seon et al., 2018, 2022), the repeatability of EFL was determined by comparing the presence and magnitude of EFL between visits 1 and 2 at peak exercise. In some cases, participants were flow‐limited at submaximal exercise intensities and/or did not reach the exact same V̇_E_ at peak exercise on both visits. Thus, we compared the presence and magnitude of EFL between visits 1 and 2 at matched levels of absolute V̇_E_ during high‐intensity exercise. To do so, we identified two exercise stages at intensities >75% of peak V̇O_2_ for each participant during which similar levels of V̇_E_ were achieved across visits 1 and 2 (mean difference: −0.72 ± 3.08 L·min^−1^). The repeatability of EFL as a binary variable was determined based on Cohen's *κ*, which is classified as “none” for a *κ* of 0.00–0.20, “minimal” for a *κ* of 0.21–0.39, “weak” for a *κ* of 0.40–0.59, “moderate” for a *κ* of 0.60–0.79, “strong” for a *κ* of 0.80–0.90, and “almost perfect” for a *κ* >0.90 (McHugh, 2012). The within‐participant repeatability of EFL magnitude as well as pulmonary function and exercise ventilatory parameters across two testing sessions was determined by computing the intraclass correlation coefficient (ICC), and was classified as “poor” for an ICC <0.50, “moderate” for an ICC of 0.50–0.75, “good” for an ICC of 0.75–0.90, and “excellent” for an ICC >0.90 (Koo & Li, 2016). Statistical analyses were performed using Python packages SciPy (Virtanen et al., 2020), Pingouin (Vallat, 2018), and SciKit‐Learn (Pedregosa et al., 2011), with the level of significance set at *p* < 0.05. All data are presented as a mean ± standard deviation or as a 95% confidence interval (CI).

## 3 RESULTS

### 3.1 Participant characteristics and pulmonary function

Table 1 summarizes the participants' physical characteristics and pulmonary function data for both study visits. There were no significant differences in physical characteristics (i.e., body mass and body mass index) or pulmonary function parameters between visit 1 and visit 2 (all *p* > 0.05).

**TABLE 1:** Participant characteristics and resting pulmonary function data.

	Visit 1 (*n* = 32)	Visit 2 (*n* = 32)	*p*
Physical characteristics			
Age, years	31.5 ± 11.9	31.5 ± 11.9	1.00
Height, cm	172 ± 8.4	172 ± 8.4	1.00
Weight, kg	74.1 ± 12.0	74.1 ± 11.9	1.00
BMI, kg⋅m^−2^	24.7 ± 2.87	24.7 ± 2.86	0.96
Spirometry			
FVC, l	4.88 ± 0.99	4.84 ± 0.94	0.06
FVC, % predicted	110 ± 13	109 ± 12	0.06
PEF, l⋅s^−1^	9.68 ± 2.16	9.95 ± 2.32	0.07
FEV_1_, l	3.94 ± 0.78	3.91 ± 0.75	0.37
FEV_1_, % predicted	105 ± 13	105 ± 11	0.31
FEV_1_/FVC, %	80.97 ± 5.13	81.19 ± 5.72	0.69
FEV_1_/FVC, % predicted	96 ± 6	96 ± 7	0.69
FEF_25–75_, l	3.89 ± 1.01	3.85 ± 1.03	0.59
FEF_25–75_, % predicted	95 ± 24	94 ± 27	0.70
IC, l	2.78 ± 0.63	2.74 ± 0.58	0.55
IC, % predicted	86 ± 15	85 ± 14	0.51
SR	1.13 ± 0.22	1.08 ± 0.24	0.03

Abbreviations: BMI, body mass index; FEF_25‐75_, forced expiratory flow from 25% to 75% of FVC; FEV_1_, forced expiratory volume in 1 s; FVC, forced vital capacity; IC, inspiratory capacity; SR, slope‐ratio index.

### 3.2 Incremental exercise responses

**FIGURE 2:**
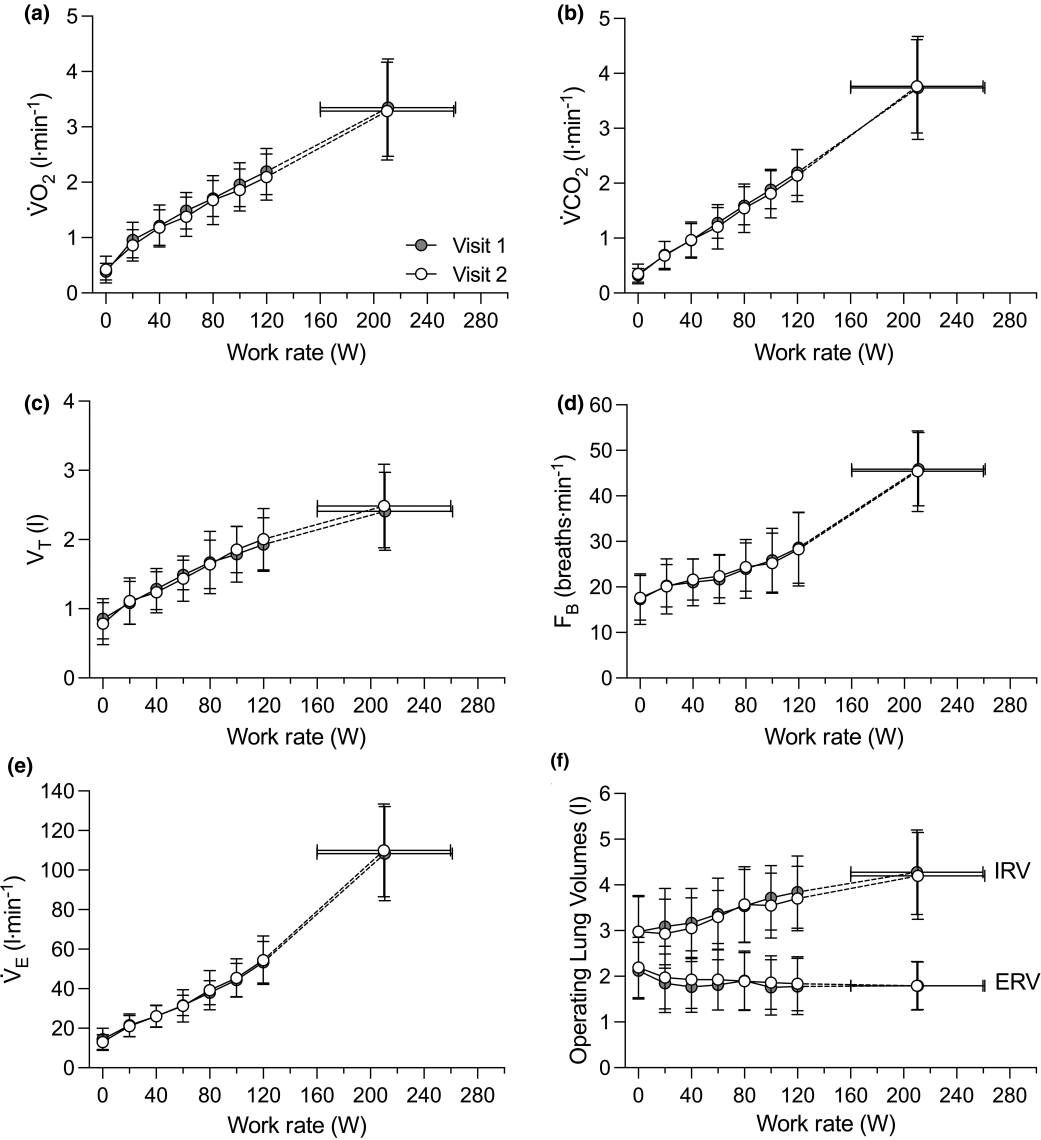
Metabolic and ventilatory responses to incremental cycle exercise during visits 1 and 2. Oxygen uptake (Panel a), carbon dioxide output (Panel b), tidal volume (Panel c), breathing frequency (Panel d), minute ventilation (Panel e), and operating lung volumes (Panel f) are plotted as a function of work rate starting a rest (i.e., 0 W) to peak exercise. The highest equivalent submaximal work rate achieved by all participants was 120 W. Data from visit 1 are shown in grey circles and the data from visit 2 are shown in white circles. Dashed lines within each visit connect the 120 W data point to the peak exercise data point. ERV, expiratory reserve volume; F_B_, breathing frequency; IRV, inspiratory reserve volume; V̇CO_2_, carbon dioxide output; V̇_E_, minute ventilation; V̇O_2_, oxygen uptake; V_T_, tidal volume.

Metabolic and ventilatory responses to incremental exercise are shown in Figure 2. There was a significant effect of work rate on V̇O_2_, carbon dioxide output, V̇_E_, breathing frequency, tidal volume, IRV, and ERV (all *p* < 0.001). However, there was neither significant effect of visit, nor the interaction between work rate and visit, on V̇O_2_, carbon dioxide output, V̇_E_, breathing frequency, tidal volume, IRV, and ERV (all *p* > 0.05), indicating that the metabolic and ventilatory responses to incremental exercise were similar between visits.

Peak exercise data are shown in Table 2. On average, participants achieved respiratory exchange ratios >1.10 and near maximum heart rates based on predicted normal values (Tanaka et al., 2001), indicating that maximal effort was exerted during both visits. There were no significant differences in cardiorespiratory data at peak exercise between visit 1 and visit 2 (all *p* > 0.05).

**TABLE 2:** Cardiorespiratory variables at peak during the incremental exercise test.

	Visit 1 (*n* = 32)	Visit 2 (*n* = 32)	*p*
Work, W	211 ± 51	210 ± 50	0.83
F_B_, breaths⋅min^−1^	46 ± 8	45 ± 9	0.70
V_T_, l	2.41 ± 0.56	2.48 ± 0.60	0.09
V_T_, % FVC	49 ± 6	51 ± 7	0.13
V̇_E_, l⋅min^−1^	108 ± 24	110 ± 24	0.59
HR, beats min^−1^	178 ± 18	177 ± 17	0.45
HR, % predicted	96 ± 7	95 ± 7	0.49
V̇O_2_, l⋅min^−1^	3.35 ± 0.88	3.28 ± 0.88	0.58
V̇CO_2_, l⋅min^−1^	3.73 ± 0.94	3.77 ± 0.85	0.71
V̇_E_/V̇O_2_	33 ± 5	35 ± 10	0.23
V̇_E_/V̇CO_2_	30 ± 4	30 ± 5	0.76
RER	1.12 ± 0.10	1.18 ± 0.21	0.25
IC, l	3.12 ± 0.64	3.14 ± 0.72	0.66
ERV, l	1.79 ± 0.52	1.79 ± 0.53	0.99
ERV, % FVC	36 ± 6	36 ± 6	0.93
IRV, l	4.20 ± 0.95	4.28 ± 0.93	0.45
IRV, % FVC	85 ± 7	90 ± 6	0.27
V̇_E,CAP_, l⋅min^−1^	172 ± 55	176 ± 48	0.51
V̇_E_/V̇_E,CAP_, %	67 ± 17	66 ± 17	0.62
V_T_/T_E_, l⋅s^−1^	3.83 ± 0.91	3.87 ± 0.84	0.87
EFL			
EFL, #	9	7	0.77
EFL, %V_T_	40 ± 24	43 ± 19	0.78

Abbreviations: EFL, expiratory flow limitation; ERV, expiratory reserve volume; F_B_, breathing frequency; FVC, forced vital capacity; HR, heart rate; IC, inspiratory capacity; IRV, inspiratory reserve volume; RER, respiratory exchange ratio; V̇CO_2_, carbon dioxide output; V̇_E_, minute ventilation; V̇_E,CAP_, ventilatory capacity; V̇_E_/V̇_E,CAP_, fractional utilization of available ventilatory capacity; V̇O_2_, oxygen uptake; V_T_, tidal volume.

### 3.3 Expiratory flow limitation

**FIGURE 3:**
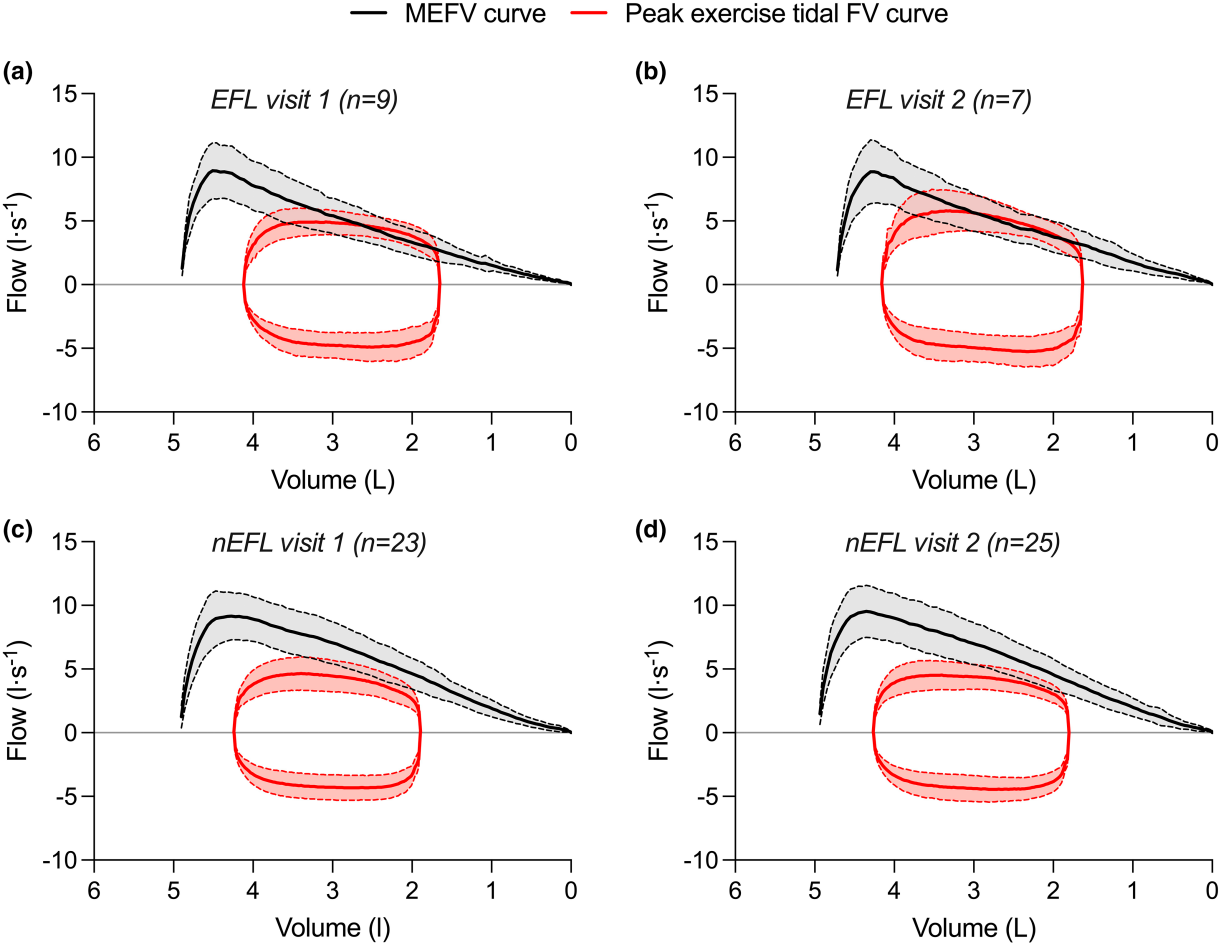
The average MEFV curve and peak exercise flow volume curve for the participants who were flow limited on visit 1 (*n* = 9) and visit 2 (*n* = 7) (a, b, respectively) and the participants who were not flow limited during either visit 1 (*n* = 23) or visit 2 (*n* = 25) (c, d, respectively). EFL, expiratory flow limitation; FV, flow‐volume; MEFV, maximum expiratory flow‐volume; FV, flow‐volume.

**FIGURE 4:**
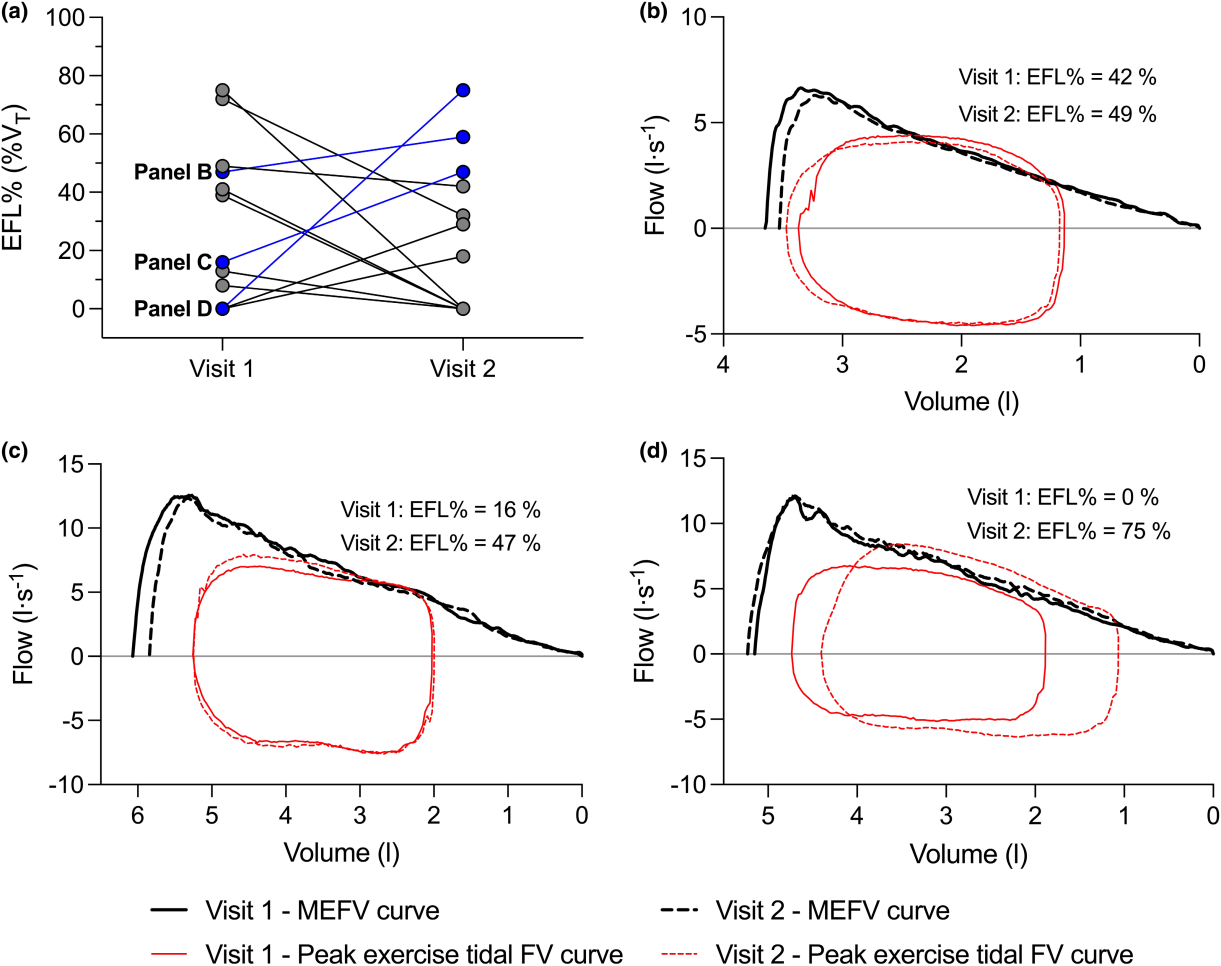
The magnitude of EFL (i.e., %EFL) at peak exercise during visit 1 and visit 2 for each individual who was flow‐limited on at least one of the two visits (a), and individual MEFV curves and peak exercise tidal flow‐volume curves during visit 1 and visit 2 for a subset of three participants (b–d). EFL, expiratory flow limitation; FV, flow‐volume; MEFV, maximum expiratory flow‐volume.

Across 736 stages (i.e., rest and individual exercise stages across two visits per participant), we found evidence of EFL in 28 cases. In total, *n* = 12 participants (38%) were flow‐limited during exercise, *n* = 9 were flow‐limited during visit 1, *n* = 7 were flow‐limited during visit 2 (Figure 3). Within the subset of participants that were flow‐limited, *n* = 5 were flow‐limited during both visits while *n* = 7 were flow‐limited during only one of the two visits. Additionally, *n* = 7 participants experienced EFL during submaximal exercise on at least one of the two visits. The average fraction of expired tidal‐volume curve that overlapped with the MEFV curve was 34 ± 21%, with 13 of the 28 cases of being below 30% of expired tidal volume. There was no significant difference in the number of participants who neither experienced EFL nor the magnitude of EFL between visit 1 and visit 2 (Table 2; both *p* > 0.05). Individual data for the magnitude of EFL for each visit in the subset of participants who experienced EFL is shown in Figure 4a along with raw data for a subset of *n* = 3 participants who differed in terms of the degree of between‐day variation in the factors involved in the assessment of EFL (Figure 4b–d).

### 3.4 Repeatability of metabolic and ventilatory parameters

Assessments of the between‐day repeatability of metabolic and ventilatory parameters during exercise are presented in Table 3. The between visit ICC for pulmonary function parameters directly involved in the assessment of EFL were 0.76–0.99 (all *p* < 0.05) indicating that the repeatability of pulmonary function parameters was “good” to “excellent.” Additionally, the variables that related to the size and shape of the MEFV curve (i.e., FVC, SR, and forced expired flow between 25% and 75% of FVC), which are indicators of ventilatory capacity, had “good” to “excellent” repeatability between visits (ICC ≥ 0.75, *p* < 0.001), and a between‐day coefficient of variation (CV) that ranged from 4% to 5%. At peak exercise, V̇O_2_ and carbon dioxide output had “moderate” to “good” repeatability between visits (ICC = 0.74 and 0.85, respectively, both *p* < 0.001), and a between‐day CV ranging from 5% to 7%. At matched levels of V̇_E_ during high‐intensity exercise, the repeatability of V̇O_2_ and carbon dioxide output improved and were considered “good” to “excellent” (ICC = 0.82 and 0.92, respectively, both *p* < 0.001) with a between‐day CV ranging from 4% to 6%. The ventilatory parameters involved in assessing EFL (i.e., tidal volume, breathing frequency, mean tidal expiratory flow, V̇_E_, IC, ERV, and IRV), which are indicators of ventilatory demand, had “good” to “excellent” repeatability at peak exercise, except for F_B_ and ERV, which had “moderate” repeatability (ICC = 0.71 and 0.60, respectively, both *p* < 0.001). The between‐day CV for ventilatory parameters at peak exercise ranged from 3% to 9%. At matched levels of V̇_E_ during high‐intensity exercise, ventilatory parameters involved in assessing EFL had “good” to “excellent” repeatability (ICC ≥ 0.75, *p* < 0.001), except for ERV which had “moderate” repeatability (ICC = 0.66, *p* < 0.001), and had a between‐day CV ranging from 3% to 9%. We also noted that the repeatability of V̇_E,CAP_ and V̇_E_/V̇_E,CAP_ was “moderate” at peak exercise (ICC = 0.74 and 0.64, respectively, both *p* < 0.001) with a between‐day CV of 7%–8%, and “good” at matched levels of V̇_E_ during high‐intensity exercise (ICC = 0.82 and 0.98, respectively, both *p* < 0.001) with a between‐day CV of 7%.

**TABLE 3:** Repeatability of metabolic and ventilatory parameters.

	CV, %	ICC	95% CI	*p*	Repeatability classification
MEFV parameters					
FVC, l	3.56	0.99	0.96–1.00	<0.001	*Excellent*
SR	5.10	0.76	0.40–0.92	<0.001	*Good*
FEF_25–75_, l⋅s^−1^	3.83	0.88	0.65–0.96	<0.001	*Good*
Peak exercise					
V̇O_2_, l⋅min^−1^	7.35	0.74	0.54–0.87	<0.001	*Moderate*
V̇CO_2_, l⋅min^−1^	5.24	0.85	0.72–0.92	<0.001	*Good*
V_T_, l	3.15	0.90	0.81–0.95	<0.001	*Excellent*
F_B_, breaths⋅min^−1^	6.00	0.71	0.48–0.85	<0.001	*Moderate*
IC, l	3.46	0.91	0.82–0.95	<0.001	*Excellent*
ERV, l	9.19	0.60	0.32–0.78	<0.001	*Moderate*
IRV, l	4.60	0.82	0.67–0.91	<0.001	*Good*
V_T_/T_E_, l⋅s^−1^	6.22	0.75	0.54–0.87	<0.001	*Good*
V̇_E_, l⋅min^−1^	6.00	0.77	0.59–0.88	<0.001	*Good*
V̇_E,CAP_, l⋅min^−1^	7.46	0.74	0.54–0.87	<0.001	*Moderate*
V̇_E_/V̇_E,CAP_, %	8.10	0.64	0.38–0.81	<0.001	*Moderate*
Matched V̇_E_					
V̇O_2_, l⋅min^−1^	5.66	0.82	0.68–0.91	<0.001	*Good*
V̇CO_2_, l⋅min^−1^	3.82	0.92	0.85–0.96	<0.001	*Excellent*
V_T_, l	3.22	0.94	0.88–0.97	<0.001	*Excellent*
F_B_, breaths⋅min^−1^	3.22	0.94	0.89–0.97	<0.001	*Excellent*
IC, l	3.74	0.93	0.86–0.96	<0.001	*Excellent*
ERV, l	9.39	0.66	0.41–0.80	<0.001	*Moderate*
IRV, l	4.57	0.97	0.95–0.99	<0.001	*Excellent*
V_T_/T_E_, l⋅s^−1^	4.34	0.93	0.85–0.96	<0.001	*Excellent*
V̇_E_, l⋅min^−1^	2.11	0.99	0.98–0.99	<0.001	*Excellent*
V̇_E,CAP_, l⋅min^−1^	7.29	0.82	0.66–0.91	<0.001	*Good*
V̇_E_/V̇_E,CAP_, %	7.12	0.88	0.77–0.94	<0.001	*Good*

Abbreviations: EFL, expiratory flow limitation; ERV, expiratory reserve volume; F_B_, breathing frequency; IC, inspiratory capacity; IRV, inspiratory reserve volume; V̇_E_, minute ventilation; V̇_E,CAP_, ventilatory capacity; V̇_E_/V̇_E,CAP_, fractional utilization of available ventilatory capacity; V_T_, tidal volume.

### 3.5 Repeatability of EFL

The between‐day repeatability of EFL is reported in Table 4. At peak exercise, the repeatability of EFL was “minimal” (*κ* = 0.34, *p* = 0.145) when assessed as a binary variable and “poor” when measured as a continuous variable (ICC = 0.34, *p* = 0.025). When examined at matched levels of V̇_E_ during high‐intensity exercise, the repeatability of EFL was “weak” when measured as a binary variable (*κ* = 0.47, *p* = 0.001) and “moderate” when measured as a continuous variable (ICC = 0.60, *p* < 0.001).

**TABLE 4:** Repeatability of EFL across all participants (*n* = 32).

	*κ*	ICC	95% CI	*p*	Repeatability classification
Peak exercise					
EFL (1,0)	0.34			0.145	*Minimal*
EFL (%V_T_ overlap)		0.34	−0.23 to 0.73	0.025	*Poor*
Matched V̇_E_ during high‐intensity exercise (i.e., >75% of peak V̇O_2_)					
EFL (1,0)	0.47			0.001	*Weak*
EFL (%V_T_ overlap)		0.60	0.33–0.78	>0.0001	*Moderate*

Abbreviations: EFL, expiratory flow limitation; V̇O_2_, oxygen uptake; V_T_, tidal volume.

## 4 DISCUSSION

### 4.1 Main findings

In this study, we assessed the repeatability of EFL during incremental exercise in a group of healthy adults by analyzing tidal expiratory flow‐volume and MEFV curves. We found that EFL occurred in relatively few participants (i.e., 38%), and that despite “moderate” to “excellent” between‐day repeatability of the individual parameters used to assess EFL, the repeatability of EFL was either “minimal” or “poor” when assessed as a binary variable and “moderate” or “weak” when assessed as a continuous variable. Our findings suggest that in healthy, 20–60‐year‐old adults of average cardiorespiratory fitness, exercise‐induced EFL occurs in less than <50% of individuals, is variable between days and does not appear to occur repeatably during incremental exercise. It should be noted that we assessed the repeatability of EFL in otherwise healthy young adults of average fitness in whom EFL is unlikely to cause exercise limitation or significantly worsen exertional dyspnea. As such, our findings do not necessarily apply to other populations such as adults above the age of 60 years, patients with respiratory disease, or endurance‐trained athletes. The between‐day variability in EFL is likely due to the dynamic nature of the ratio between ventilatory capacity and ventilatory demand throughout incremental exercise and the inherent limitations associated with the assessment of EFL based on the comparison of tidal expiratory flow‐volume and MEFV curves.

### 4.2 Prevalence and mechanisms for EFL

In healthy adults, ventilatory capacity typically exceeds ventilatory demand throughout low‐ to moderate‐intensity exercise such that the vast majority of individuals do not experience EFL (Molgat‐Seon et al., 2018). However, the fractional utilization of ventilatory capacity increases as a function of exercise intensity, with some individuals reaching their maximum capacity for expiratory flow generation during high‐intensity exercise. Indeed, EFL occurs during maximal exercise in 44%–62% of healthy adults (Aaron et al., 1992; Dominelli et al., 2011, 2015; Mann et al., 2020; Molgat‐Seon et al., 2022; Smith et al., 2014). The range of the frequencies of EFL at peak exercise across these studies likely reflects the different techniques used to assess EFL, the average aerobic fitness of the participants, and/or differences in the exercise modality. We noted that 38% of participants experienced EFL during exercise on at least one occasion (Table 2), which is similar, albeit slightly lower, than the frequency of EFL reported in previous studies involving healthy adults with similar levels of cardiorespiratory fitness. We also noted that within the subset of participants that experienced EFL at some point during exercise, that 34 ± 21% of expired tidal volume overlapped with the MEFV curve, which is considered “mild” (i.e., <50% EFL) in terms of ventilatory constraint (Johnson et al., 1999).

The detection of EFL based on the analysis of tidal expiratory flow‐volume and MEFV curves can be used during cardiopulmonary exercise testing as an index of mechanical ventilatory constraint in addition to traditional measures of breathing reserve (e.g., the ratio of V̇_E_ and maximum voluntary ventilation). Clearly, assessments of EFL provide important information related to the degree of mechanical ventilatory constraint during exercise (Johnson et al., 1999), but increasing the use of EFL measures during cardiopulmonary exercise testing requires that the repeatability of the technique be determined.

### 4.3 Repeatability of EFL

We noted that the repeatability of EFL was “minimal” to “poor” when assessed as a binary variable and “moderate” to “weak” when assessed as a continuous variable (Table 4). Variability in the presence and magnitude of EFL could be due to several factors, including between‐day variation in ventilatory capacity, ventilatory demand, or the inherent error of the method used to assess EFL.

There is clear evidence of short‐term variation in pulmonary function within an individual. For example, diurnal variation in measures of pulmonary function have been reported (Medarov et al., 2008); however, the consensus is that the extent of variation is relatively small. Although there are no generally accepted standards for between‐day repeatability of spirometry parameters (e.g., FVC, and forced expired flow from 25% to 75% of FVC), the between‐day coefficient of variation in healthy adults is ~3%–13% (Pennock et al., 1981; Rozas & Goldman, 1982). Similarly, SR which is a measure describing the shape of the MEFV curve, is highly repeatable in healthy adults (Tien et al., 1979). When used in combination, measures of FVC, forced expired flow from 25% to 75% of FVC, and SR provide a detailed description of an individual's ventilatory capacity, and we noted that the repeatability of these measures was “good” to “excellent” between days (Table 3). As such, the low degree of repeatability of EFL is unlikely to be explained exclusively by between‐day variability in measures of ventilatory capacity.

As expected, the V̇O_2_ and carbon dioxide output associated with a given absolute work rate was similar between days (Figure 2a,b). Ventilatory demand is directly linked to whole‐body metabolism and can be assessed based on V̇_E_. Assessments of tidal volume, breathing frequency, mean expiratory flow, and operating lung volumes provide detailed information regarding the breathing pattern used to achieve a given V̇_E_. At peak exercise, the repeatability of indices of ventilatory demand were either “moderate,” “good,” or “excellent” (Table 3). However, comparisons at peak exercise may not always be appropriate, particularly if an individual does not reach the exact same time or stage on both days. Thus, we assessed the between‐day repeatability of EFL at similar levels of V̇_E_ achieved on both days at exercise intensities >75% of peak V̇O_2_. In doing so, we were able to account for between‐visit differences in peak V̇_E_ and analyze the repeatability of EFL by minimizing variability in ventilatory demand. The repeatability of EFL at similar levels of V̇_E_ during high‐intensity exercise improved slightly compared to peak exercise but was still considered “weak” and “moderate” for EFL assessed as a binary variable and as a continuous variable, respectively (Table 4).

There are several methods used to assess EFL during tidal breathing, each with varying degrees of precision and technical difficulty (Koulouris & Hardavella, 2011). Overlaying tidal expiratory flow‐volume and MEFV curves is the most ubiquitous technique (Johnson et al., 1999; Koulouris & Hardavella, 2011), presumably due to its relative simplicity and feasibility in comparison to other techniques. However, this method has well‐documented limitations, including the reliance on participant cooperation, the confounding influence of thoracic gas compression and exercise‐induced changes in bronchial tone, potential errors due to alignment of the tidal and maximal expiratory flow‐volume curves, as well as differences in the volume and time history, respiratory mechanics, and time constants between the tidal and MEFV curves (Koulouris & Hardavella, 2011). Thus, it is possible that the between‐day variability in EFL could be explained by the inherent error associated with this method, which likely reflects the combined error of each factor involved in the assessed EFL (Table 3). Although the error of each individual factor (e.g., IC) may be small, the summation of errors for all factors involved in the assessment of EFL may be sufficient to erode the repeatability of EFL. For example, the between‐day CV for all parameters involved in the assessment of EFL ranged from 3% to 9% (Table 3), small variations in IC, tidal flow‐volume and MEFV curve flow between visits result in substantial differences in the presence and/or magnitude of EFL during high‐intensity exercise. Indeed, in a subset of participants who differed in terms of the degree of between‐day variation in the factors involved in the assessment of EFL, the assessment of EFL was directly impacted (Figure 4b–d).

Overall, we ascribe our findings to the impact of slight variations in the measures of ventilatory demand and/or ventilatory capacity on the assessment of EFL. Indeed, the method we used to assess EFL is sensitive to small errors in MEFV parameters, tidal volume, breathing frequency, mean expiratory flow, and operating lung volumes, which can affect the assessment of EFL individually or in combination (Figure 4). We emphasize that our findings should not necessarily be viewed as critique of the method we used to assess EFL, but rather as an indication of the limitations associated with applying the method to perform repeated measures of EFL during exercise in healthy adults and viewing EFL as an “all‐or‐none” phenomenon (Babb, 2013). Indeed, in healthy adults, EFL can be avoided with slight modifications in breathing pattern due to their large ventilatory capacity (Guenette et al., 2007). When coupled with the fact that, when combined, even slight errors in tidal volume, breathing frequency, tidal expiratory flow, IC, and/or MEFV curve parameters have a substantial impact on the presence or magnitude of EFL (Figure 4), it seems unsurprising that EFL would not be repeatable within a healthy individual during incremental exercise.

### 4.4 Limitations

Our study has some important limitations that merit discussion. First, we assessed EFL by analyzing tidal expiratory flow‐volume and MEFV curves and did not determine whether participants experienced EFL based on isovolume relationships between flow and transpulmonary pressure, which is viewed as the most direct method of assessing EFL. Our goal was to determine the repeatability of EFL when assessed using tidal expiratory flow‐volume and MEFV curve analysis, not to compare this method to a gold standard. Second, the frequency of EFL during exercise within our sample was slightly lower than previous studies. This may be due to the range of pulmonary function parameters and/or cardiorespiratory fitness of the participants in our sample, and we acknowledge that it is possible that the repeatability of exercise‐induced EFL may be higher in other groups of healthy individuals, such as those with high cardiorespiratory fitness who utilize a greater fraction of their available ventilatory capacity during high‐intensity exercise. Additional studies involving groups of individuals with a higher propensity toward exercise‐induced EFL (e.g., older adults, patients with chronic respiratory disease, and endurance‐training athletes) is required in order to further characterize the repeatability of EFL during exercise. Third, we did not include measures of esophageal pressure in our study, which makes the quantification of effort during IC maneuvers difficult. Nevertheless, we followed established guidelines for IC measurement (Guenette et al., 2013), and are therefore confident in the validity of our IC measures. Finally, we only included healthy adults of average cardiorespiratory fitness and our results do not necessarily extend to older adults, patients with chronic respiratory disease, or endurance‐trained athletes.

### 4.5 Implications

The results of our study indicate that in healthy adults of average fitness, EFL may be more of a variable phenomenon than previously thought. According to the concept of “impending flow‐limitation,” dynamic compression of the airways can occur when expiratory flow approaches, but does not reach, maximal expiratory flow (Babb, 2013). It follows that in certain circumstances, EFL need not be present in order to adversely affect the mechanical ventilatory response to exercise, which is important since we found that the assessment of EFL by overlaying expiratory tidal expiratory flow‐volume and MEFV curves has poor repeatability during exercise in healthy adults. Thus, variability in the presence and magnitude of EFL may not necessarily imply that there is corresponding variability in the physiological consequences associated with airflow limitation. We propose that measures of EFL should be interpreted in the context of other indices of mechanical ventilatory constraint, such as operating lung volumes and the work of breathing, when assessing the ventilatory response to exercise in healthy adults.

We also assessed the repeatability of V̇_E_/V̇_E,CAP_, a continuous measure of the ventilatory demand‐capacity ratio that accounts for the effect of changes in operating lung volumes on maximal expiratory flows (Johnson et al., 1999). While this technique is not new, it is relatively underutilized. Given that the mechanical consequences of EFL can occur prior to overt EFL (Babb, 2013), a technique that provides a continuous measure of the ventilatory demand‐capacity ratio, such as V̇_E_/V̇_E,CAP_, may be superior to measures of the presence and magnitude of EFL (Babb & Rodarte, 1993). We noted that the between‐day repeatability of V̇_E_/V̇_E,CAP_ was “moderate” to “good” (Table 3). It is possible that V̇_E_/V̇_E,CAP_ could be used as an alternative to measures of EFL during exercise that provides similar information regarding the degree of mechanical ventilatory constraint but with greater repeatability; however, this requires additional research.

## 5 CONCLUSION

We assessed the repeatability of EFL during exercise in a group of healthy adults who performed incremental exercise. Our findings indicate that despite “good” to “excellent” between‐day repeatability of the individual parameters used to assess EFL based on analysis of tidal expiratory flow‐volume and MEFV curves (i.e., operating lung volumes, tidal expiratory flow‐volume curves, and the MEFV curve), the repeatability of EFL was either “minimal” or “poor” when assessed as a binary variable and “moderate” or “weak” when assessed as a continuous variable. Although this method of assessing EFL during exercise is widely used, it may not be appropriate for examining EFL when repeated measures are involved, which highlights the need for the development of novel indicators of mechanical ventilatory constraint that can be applied repeatably during cardiopulmonary exercise testing.

## FUNDING INFORMATION

Funding for this study was provided by the Natural Sciences and Engineering Research Council of Canada (NSERC; RGPIN‐2020‐06564), Research Manitoba (5409), and the University of Winnipeg. JRD was supported by an Undergraduate Student Research Award (USRA) from NSERC.

## CONFLICT OF INTEREST STATEMENT

The authors declare no conflicts of interest.

## ETHICS STATEMENT

The study was approved by the University of Winnipeg's Human Research Ethics Board (#HE17119) and was conducted in accordance with the ethical principles of the Declaration of Helsinki.

## Supporting information


Data S1.

## Data Availability

Data generated or analyzed during this study are provided in full within the published article and are available from the corresponding author upon reasonable request.

## References

[phy270068-bib-0001] Aaron, E. A. , Johnson, B. D. , Seow, C. K. , & Dempsey, J. A. (1992). Oxygen cost of exercise hyperpnea: Measurement. Journal of Applied Physiology, 72, 1810. 1601790 10.1152/jappl.1992.72.5.1810

[phy270068-bib-0002] American Thoracic Society, and American College of Chest Physicians . (2003). ATS/ACCP Statement on cardiopulmonary exercise testing. American Journal of Respiratory and Critical Care Medicine, 167, 211–277. 12524257 10.1164/rccm.167.2.211

[phy270068-bib-0003] Babb, T. G. (2013). Exercise ventilatory limitation: The role of expiratory flow limitation. Exercise and Sport Sciences Reviews, 41, 11–18. 23038244 10.1097/JES.0b013e318267c0d2 PMC3529766

[phy270068-bib-0004] Babb, T. G. , & Rodarte, J. R. (1993). Estimation of ventilatory capacity during submaximal exercise. Journal of Applied Physiology, 74, 2016–2022. 8514724 10.1152/jappl.1993.74.4.2016

[phy270068-bib-0005] Bartlett, R. G., Jr. , Phillips, N. E. , & Wolski, G. (1963). Maximum voluntary ventilation prediction from the velocity‐volume loop. Diseases of the Chest, 43, 382–392. 13969714 10.1378/chest.43.4.382

[phy270068-bib-0006] Chen, R. , Chen, L. , Chen, R. C. , & Chen, X. (2010). Reproducibility of the negative expiratory pressure technique in detecting expiratory flow limitation on chronic obstructive pulmonary disease patients. Zhonghua Liu Xing Bing Xue Za Zhi, 31, 1397–1399. 21223672

[phy270068-bib-0007] Dempsey, J. A. , La Gerche, A. , & Hull, J. H. (2020). Is the healthy respiratory system built just right, overbuilt, or underbuilt to meet the demands imposed by exercise? Journal of Applied Physiology, 129, 1235–1256. 32790594 10.1152/japplphysiol.00444.2020 PMC7864246

[phy270068-bib-0008] Dominelli, P. B. , Foster, G. E. , Dominelli, G. S. , Henderson, W. R. , Koehle, M. S. , McKenzie, D. C. , & Sheel, A. W. (2013). Exercise‐induced arterial hypoxaemia and the mechanics of breathing in healthy young women. The Journal of Physiology, 591, 3017–3034. 23587886 10.1113/jphysiol.2013.252767 PMC3832117

[phy270068-bib-0009] Dominelli, P. B. , Guenette, J. A. , Wilkie, S. S. , Foster, G. E. , & Sheel, A. W. (2011). Determinants of expiratory flow limitation in healthy women during exercise. Medicine and Science in Sports and Exercise, 43, 1666–1674. 21364489 10.1249/MSS.0b013e318214679d

[phy270068-bib-0010] Dominelli, P. B. , Molgat‐Seon, Y. , Bingham, D. , Swartz, P. M. , Road, J. D. , Foster, G. E. , & Sheel, A. W. (2015). Dysanapsis and the resistive work of breathing during exercise in healthy men and women. Journal of Applied Physiology, 119, 1105–1113. 26359483 10.1152/japplphysiol.00409.2015 PMC4816413

[phy270068-bib-0011] Dominelli, P. B. , Molgat‐Seon, Y. , Foster, G. E. , Dominelli, G. S. , Haverkamp, H. C. , Henderson, W. R. , & Sheel, A. W. (2016). Quantifying the shape of maximal expiratory flow‐volume curves in healthy humans and asthmatic patients. Respiratory Physiology & Neurobiology, 220, 46–53. 26388199 10.1016/j.resp.2015.09.007 PMC6238952

[phy270068-bib-0012] Dominelli, P. B. , Render, J. N. , Molgat‐Seon, Y. , Foster, G. E. , & Sheel, A. W. (2014). Precise mimicking of exercise hyperpnea to investigate the oxygen cost of breathing. Respiratory Physiology & Neurobiology, 201, 15–23. 24981705 10.1016/j.resp.2014.06.010

[phy270068-bib-0013] Dominelli, P. B. , & Sheel, A. W. (2012). Experimental approaches to the study of the mechanics of breathing during exercise. Respiratory Physiology & Neurobiology, 180, 147–161. 22019486 10.1016/j.resp.2011.10.005

[phy270068-bib-0014] Graham, B. L. , Steenbruggen, I. , Miller, M. R. , Barjaktarevic, I. Z. , Cooper, B. G. , Hall, G. L. , Hallstrand, T. S. , Kaminsky, D. A. , McCarthy, K. , McCormack, M. C. , Oropez, C. E. , Rosenfeld, M. , Stanojevic, S. , Swanney, M. P. , & Thompson, B. R. (2019). Standardization of spirometry 2019 update. An official American Thoracic Society and European Respiratory Society technical Statement. American Journal of Respiratory and Critical Care Medicine, 200, e70–e88. 31613151 10.1164/rccm.201908-1590ST PMC6794117

[phy270068-bib-0015] Grimby, G. , Saltin, B. , & Wilhelmsen, L. (1971). Pulmonary flow‐volume and pressure‐volume relationship during submaximal and maximal exercise in young well‐trained men. Bulletin De Physio‐Pathologie Respiratoire, 7, 157–172. 5113052

[phy270068-bib-0016] Guenette, J. A. , Chin, R. C. , Cory, J. M. , Webb, K. A. , & O'Donnell, D. E. (2013). Inspiratory capacity during exercise: Measurement, analysis, and interpretation. Pulmonary Medicine, 2013, 956081. 23476765 10.1155/2013/956081 PMC3582111

[phy270068-bib-0017] Guenette, J. A. , Dominelli, P. B. , Reeve, S. S. , Durkin, C. M. , Eves, N. D. , & Sheel, A. W. (2010). Effect of thoracic gas compression and bronchodilation on the assessment of expiratory flow limitation during exercise in healthy humans. Respiratory Physiology & Neurobiology, 170, 279–286. 20138157 10.1016/j.resp.2010.01.017

[phy270068-bib-0018] Guenette, J. A. , Witt, J. D. , McKenzie, D. C. , Road, J. D. , & Sheel, A. W. (2007). Respiratory mechanics during exercise in endurance‐trained men and women. The Journal of Physiology, 581, 1309–1322. 17412775 10.1113/jphysiol.2006.126466 PMC2170830

[phy270068-bib-0019] Hyatt, R. E. (1961). The interrelationships of pressure, flow, and volume during various respiratory maneuvers in normal and emphysematous subjects. The American Review of Respiratory Disease, 83, 676–683. 13717137 10.1164/arrd.1961.83.5.676

[phy270068-bib-0020] Hyatt, R. E. (1983). Expiratory flow limitation. Journal of Applied Physiology: Respiratory, Environmental and Exercise Physiology, 55, 1–7. 6350246 10.1152/jappl.1983.55.1.1

[phy270068-bib-0021] Iandelli, I. , Aliverti, A. , Kayser, B. , Dellaca, R. , Cala, S. J. , Duranti, R. , Kelly, S. , Scano, G. , Sliwinski, P. , Yan, S. , Macklem, P. T. , & Pedotti, A. (2002). Determinants of exercise performance in normal men with externally imposed expiratory flow limitation. Journal of Applied Physiology, 92, 1943–1952. 11960944 10.1152/japplphysiol.00393.2000

[phy270068-bib-0022] Johnson, B. D. , & Dempsey, J. A. (1991). Demand vs. capacity in the aging pulmonary system. Exercise and Sport Sciences Reviews, 19, 171–210. 1936085

[phy270068-bib-0023] Johnson, B. D. , Weisman, I. M. , Zeballos, R. J. , & Beck, K. C. (1999). Emerging concepts in the evaluation of ventilatory limitation during exercise: The exercise tidal flow‐volume loop. Chest, 116, 488–503. 10453881 10.1378/chest.116.2.488

[phy270068-bib-0024] Koo, T. K. , & Li, M. Y. (2016). A guideline of selecting and reporting intraclass correlation coefficients for reliability research. Journal of Chiropractic Medicine, 15, 155–163. 27330520 10.1016/j.jcm.2016.02.012 PMC4913118

[phy270068-bib-0025] Koulouris, N. G. , & Hardavella, G. (2011). Physiological techniques for detecting expiratory flow limitation during tidal breathing. European Respiratory Review, 20, 147–155. 21881143 10.1183/09059180.00001911 PMC9584109

[phy270068-bib-0026] Mann, L. M. , Granger, E. A. , Chan, J. S. , Yu, A. , Molgat‐Seon, Y. , & Dominelli, P. B. (2020). Minimizing airflow turbulence in women lowers the work of breathing to levels similar to men. Journal of Applied Physiology, 129, 410–418. 32702273 10.1152/japplphysiol.00347.2020 PMC7473950

[phy270068-bib-0027] McHugh, M. L. (2012). Interrater reliability: The kappa statistic. Biochemia Medica, 22, 276–282. 23092060 PMC3900052

[phy270068-bib-0028] Medarov, B. I. , Pavlov, V. A. , & Rossoff, L. (2008). Diurnal variations in human pulmonary function. International Journal of Clinical and Experimental Medicine, 1, 267–273. 19079662 PMC2592592

[phy270068-bib-0029] Molgat‐Seon, Y. , Dominelli, P. B. , Peters, C. M. , Kipp, S. , Welch, J. F. , Parmar, H. R. , Rabbani, T. , Mann, L. M. , Grift, G. O. , Guenette, J. A. , & Sheel, A. W. (2022). Predictors of expiratory flow limitation during exercise in healthy males and females. Medicine and Science in Sports and Exercise, 54, 1428–1436. 35438665 10.1249/MSS.0000000000002938

[phy270068-bib-0030] Molgat‐Seon, Y. , Dominelli, P. B. , Ramsook, A. H. , Schaeffer, M. R. , Molgat Sereacki, S. , Foster, G. E. , Romer, L. M. , Road, J. D. , Guenette, J. A. , & Sheel, A. W. (2018). The effects of age and sex on mechanical ventilatory constraint and dyspnea during exercise in healthy humans. Journal of Applied Physiology, 124, 1092–1106. 29357513 10.1152/japplphysiol.00608.2017 PMC5972460

[phy270068-bib-0031] Pedregosa, F. , Varoquaux, G. , Gramfort, A. , Michel, V. , Thirion, B. , Grisel, O. , Blondel, M. , Prettenhofer, P. , Weiss, R. , Dubourg, V. , Vanderplas, J. , Passos, A. , Cournapeau, D. , Brucher, M. , Perrot, M. , & Duchesnay, É. (2011). Scikit‐learn: Machine learning in python. Journal of Machine Learning Research, 12, 2825–2830.

[phy270068-bib-0032] Pellegrino, R. , Brusasco, V. , Rodarte, J. R. , & Babb, T. G. (1993). Expiratory flow limitation and regulation of end‐expiratory lung volume during exercise. Journal of Applied Physiology, 74, 2552–2558. 8335591 10.1152/jappl.1993.74.5.2552

[phy270068-bib-0033] Pennock, B. E. , Rogers, R. M. , & McCaffree, D. R. (1981). Changes in measured spirometric indices. What is significant? Chest, 80, 97–99. 7249720 10.1378/chest.80.1.97

[phy270068-bib-0034] Peters, C. M. , Dempsey, J. A. , Hopkins, S. R. , & Sheel, A. W. (2023). Is the lung built for exercise? Advances and unresolved questions. Medicine and Science in Sports and Exercise, 55, 2143–2159. 37443459 10.1249/MSS.0000000000003255 PMC11186580

[phy270068-bib-0035] Quanjer, P. H. , Stanojevic, S. , Cole, T. J. , Baur, X. , Hall, G. L. , Culver, B. H. , Enright, P. L. , Hankinson, J. L. , Ip, M. S. , Zheng, J. , Stocks, J. , & Initiative, E. R. S. G. L. F. (2012). Multi‐ethnic reference values for spirometry for the 3‐95‐yr age range: the global lung function 2012 equations. The European Respiratory Journal, 40, 1324. 22743675 10.1183/09031936.00080312 PMC3786581

[phy270068-bib-0036] Rozas, C. J. , & Goldman, A. L. (1982). Daily spirometric variability: Normal subjects and subjects with chronic bronchitis with and without airflow obstruction. Archives of Internal Medicine, 142, 1287–1291. 7092446 10.1001/archinte.142.7.1287

[phy270068-bib-0037] Smith, J. R. , Kurti, S. P. , Meskimen, K. , & Harms, C. A. (2017). Expiratory flow limitation and operating lung volumes during exercise in older and younger adults. Respiratory Physiology & Neurobiology, 240, 26–31. 28232071 10.1016/j.resp.2016.12.016

[phy270068-bib-0038] Smith, J. R. , Rosenkranz, S. K. , & Harms, C. A. (2014). Dysanapsis ratio as a predictor for expiratory flow limitation. Respiratory Physiology & Neurobiology, 198, 25–31. 24726854 10.1016/j.resp.2014.04.001

[phy270068-bib-0039] Stickland, M. K. , Neder, J. A. , Guenette, J. A. , O'Donnell, D. E. , & Jensen, D. (2022). Using cardiopulmonary exercise testing to understand dyspnea and exercise intolerance in respiratory disease. Chest, 161, 1505–1516. 35065052 10.1016/j.chest.2022.01.021

[phy270068-bib-0040] Stucky, F. , Uva, B. , Kayser, B. , & Aliverti, A. (2023). Blood shifts between body compartments during submaximal exercise with induced expiratory flow limitation in healthy humans. The Journal of Physiology, 601, 227–244. 36367253 10.1113/JP283176

[phy270068-bib-0041] Tanaka, H. , Monahan, K. D. , & Seals, D. R. (2001). Age‐predicted maximal heart rate revisited. Journal of the American College of Cardiology, 37, 153–156. 11153730 10.1016/s0735-1097(00)01054-8

[phy270068-bib-0042] Tantucci, C. (2013). Expiratory flow limitation definition, mechanisms, methods, and significance. Pulm Med, 2013, 749860. 23606962 10.1155/2013/749860 PMC3625607

[phy270068-bib-0043] Tien, Y. K. , Elliott, E. A. , & Mead, J. (1979). Variability of the configuration of maximum expiratory flow‐volume curves. Journal of Applied Physiology: Respiratory, Environmental and Exercise Physiology, 46, 565–570. 438028 10.1152/jappl.1979.46.3.565

[phy270068-bib-0044] Vallat, R. (2018). Pingouin: Statistics in Python. Journal of Open Source Software, 3, 1026.

[phy270068-bib-0045] Virtanen, P. , Gommers, R. , Oliphant, T. E. , Haberland, M. , Reddy, T. , Cournapeau, D. , Burovski, E. , Peterson, P. , Weckesser, W. , Bright, J. , van der Walt, S. J. , Brett, M. , Wilson, J. , Millman, K. J. , Mayorov, N. , Nelson, A. R. J. , Jones, E. , Kern, R. , Larson, E. , … SciPy, C. (2020). SciPy 1.0: Fundamental algorithms for scientific computing in python. Nature Methods, 17, 261–272. 32015543 10.1038/s41592-019-0686-2 PMC7056644

[phy270068-bib-0046] Walker, R. , Paratz, J. , & Holland, A. E. (2007). Reproducibility of the negative expiratory pressure technique in COPD. Chest, 132, 471–476. 17573493 10.1378/chest.07-0062

