# Supplementary material for: Assessing the repeatability of expiratory flow limitation during incremental exercise in healthy adults
Source: Physiol Rep. 2024 Oct 2;12(19):e70068. doi: 10.14814/phy2.70068 (PMC11446834; doi:10.14814/phy2.70068)
Supplement: Supplementary file 1 — Data S1. [file PHY2-12-e70068-s001.docx]

**SUPPLEMENTARY MATERIAL**

**Assessing the repeatability of expiratory flow limitation during incremental exercise in healthy adults**

Jack R. Dunsford^1^, Jasvir K. Dhaliwal^1^, Gracie O. Grift^1^, Robert Pryce^1^, Paolo B. Dominelli^2^, and Yannick Molgat-Seon^1,3^*****

^1^ Department of Kinesiology and Applied Health, University of Winnipeg, Winnipeg, Manitoba, Canada, R3B 2E9

^2^ Department of Kinesiology and Health Sciences, University of Waterloo, Waterloo, Ontario,

Canada, N2L 3G1

^3^ Centre for Heart and Lung Innovation, St. Paul’s Hospital, Vancouver, British Columbia, Canada, V6Z 1Y6

**Running Title**: Expiratory flow limitation during exercise

*** Corresponding Author**: Yannick Molgat-Seon, Ph.D.

515 Portage Ave.

Department of Kinesiology and Applied Health

Winnipeg, Manitoba, Canada, R3B 0E9

E-mail: y.molgat-seon@uwinnipeg.ca

Phone: +1 (204) 988-7640

**SUPPLEMENTARY RESULTS**

Participant characteristics and pulmonary function:

**Table S1** summarizes the physical characteristics and pulmonary function data for both study visits in the subset of n=12 participants (n=8 males, n=4 females) that experienced EFL during exercise during at least one of the two visits. There were no significant differences in physical characteristics (*i.e.*, body mass and body mass index) or pulmonary function parameters between visit 1 and visit 2 (all *p*>0.05).

Incremental exercise responses:

Peak exercise data for the subset of n=12 participants that experienced EFL during exercise during at least one of the two visits are shown in **Table S2**. On average, participants achieved respiratory exchange ratios >1.10 and near maximum heart rates based on predicted normal values (1), indicating that maximal effort was exerted during both visits. Absolute V_T_ was significantly lower during visit 1 than visit 2 (*p*=0.04); however, there were no significant differences in in any other ventilatory, metabolic, or cardiovascular parameters at peak exercise between visit 1 and visit 2 (all *p*>0.05).

Repeatability of metabolic and ventilatory parameters:

Assessments of the between-day repeatability of metabolic and ventilatory parameters during exercise in the n=12 participants who experienced EFL on at least one of the two visits are presented in **Table S3**. The variables that related to the size and shape of the MEFV curve (*i.e.*, FVC, SR, and forced expired flow between 25 and 75% of FVC), which are indicators of ventilatory capacity, had ‘good’ to ‘excellent’ repeatability between visits (ICC≥0.84, *p*<0.001), and a between-day coefficient of variation (CV) that ranged from 2-5 %. At peak exercise, V̇O_2_ and carbon dioxide output had ‘moderate’ to ‘good’ repeatability between visits (ICC=0.79 and 0.81, respectively, both *p*<0.001), and a between-day CV ranging from 15-16 %. At matched levels of V̇_E_ during high-intensity exercise, the repeatability of V̇O_2_ and carbon dioxide output improved and were considered ‘good’ (ICC= 0.78 and 0.83, respectively, both *p*<0.001) with a between-day CV ranging from 5-6 %. Tidal volume, mean tidal expiratory flow, V̇_E_, and IC had ‘good’ to ‘excellent’ repeatability at peak exercise (ICC≥0.78; all *p*<0.001), whereas F_B_, ERV and IRV had ‘moderate’ repeatability (ICC≥0.57; all *p*<0.001). The between-day CV for ventilatory parameters at peak exercise ranged from 5-15 %. At matched levels of V̇_E_ during high-intensity exercise, ventilatory parameters involved in assessing EFL had ‘good’ to ‘excellent’ repeatability (ICC≥0.75, *p*<0.001), except for ERV which had ‘moderate’ repeatability (ICC=0.70, *p*<0.001), and had a between-day CV ranging from 3-9 %. The repeatability of V̇_E, CAP_ and V̇_E_/V̇_E, CAP_ was ‘poor’ at peak exercise (ICC=0.46 and 0.09, respectively, both *p*<0.001) with a between-day CV of 10-19 %, but ‘good’ at matched levels of V̇_E_ during high-intensity exercise (ICC=0.78 and 0.82, respectively, both *p*<0.001) with a between-day CV of 7 %.

**REFERENCE**

1. **Tanaka H, Monahan KD, and Seals DR**. Age-predicted maximal heart rate revisited. *J Am Coll Cardiol* 37: 153-156, 2001.

**SUPPLEMENTARY TABLES**

**Table S1**. Participant characteristics and resting pulmonary function data on visit 1 and visit 2 in the participants who experienced EFL during at least 1 of the 2 visits.

|  | Visit 1 (n=12) | | | Visit 2 (n=12) | | | *p* |
| --- | --- | --- | --- | --- | --- | --- | --- |
| *Physical Characteristics* |  |  |  |  |  |  |  |
| Age, y | 29.6 | ± | 8.1 | 29.6 | ± | 8.10 | 1.00 |
| Height, cm | 173 | ± | 7.5 | 173 | ± | 7.5 | 1.00 |
| Weight, kg | 72.5 | ± | 8.9 | 72.6 | ± | 8.7 | 0.59 |
| BMI, kg⋅m^-2^ | 24.2 | ± | 2.9 | 24.3 | ± | 2.84 | 0.60 |
| *Spirometry* |  |  |  |  |  |  |  |
| FVC, l | 4.90 | ± | 0.97 | 4.90 | ± | 0.96 | 0.92 |
| FVC, % predicted | 108 | ± | 13 | 108 | ± | 11 | 0.89 |
| PEF, l⋅s^-1^ | 9.40 | ± | 2.29 | 9.97 | ± | 2.62 | 0.07 |
| FEV_1_, l | 3.75 | ± | 0.69 | 3.81 | ± | 0.77 | 0.31 |
| FEV_1_, % predicted | 89 | ± | 29 | 99 | ± | 8 | 0.24 |
| FEV_1_/FVC, % | 76.7 | ± | 4.00 | 78.0 | ± | 5.01 | 0.20 |
| FEV_1_/FVC, % predicted | 91 | ± | 6 | 92 | ± | 7 | 0.19 |
| FEF_25-75_, l | 3.17 | ± | 0.67 | 3.19 | ± | 0.74 | 0.85 |
| FEF_25-75_, % predicted | 74 | ± | 11 | 75 | ± | 14 | 0.74 |
| IC, l | 2.64 | ± | 0.57 | 2.71 | ± | 0.60 | 0.65 |
| IC, % predicted | 81 | ± | 13 | 82 | ± | 12 | 0.69 |
| SR | 1.24 | ± | 0.20 | 1.22 | ± | 0.26 | 0.63 |

*Abbreviations:* BMI, body mass index; FEF_25-75_, forced expiratory flow from 25-75% of FVC; FEV_1_, forced expiratory volume in 1 second; FVC, forced vital capacity; IC, inspiratory capacity; SR, slope-ratio index.

**Table S2**. Cardiorespiratory variables at peak during the incremental exercise tests performed on visit 1 and visit 2 in the participants who experienced EFL during at least 1 of the 2 visits.

|  | Visit 1 (n=12) | | | Visit 2 (n=12) | | | *p* |
| --- | --- | --- | --- | --- | --- | --- | --- |
| Work, W | 218 | ± | 37 | 220 | ± | 42 | 0.75 |
| F_B_, breaths⋅min^-1^ | 48 | ± | 5 | 45 | ± | 9 | 0.14 |
| V_T_, l | 2.42 | ± | 0.50 | 2.63 | ± | 0.58 | 0.04* |
| V_T_, % FVC | 50 | ± | 6 | 53 | ± | 7 | 0.06 |
| V̇_E_, l⋅min^-1^ | 115 | ± | 22 | 116 | ± | 28 | 0.77 |
| HR, beats⋅min^-1^ | 181 | ± | 14 | 181 | ± | 15 | 0.68 |
| HR, % predicted | 97 | ± | 6 | 96 | ± | 6 | 0.67 |
| V̇O_2_, l⋅min^-1^ | 3.29 | ± | 0.86 | 3.32 | ± | 0.90 | 0.88 |
| V̇CO_2_, l⋅min^-1^ | 3.77 | ± | 0.93 | 3.88 | ± | 0.98 | 0.52 |
| V̇_E_/V̇O_2_ | 36 | ± | 6 | 37 | ± | 10 | 0.79 |
| V̇_E_/V̇CO_2_ | 31 | ± | 4 | 31 | ± | 8 | 0.92 |
| RER | 1.15 | ± | 0.05 | 1 | ± | 0 | 0.14 |
| IC, l | 3.16 | ± | 0.70 | 3.21 | ± | 0.69 | 0.58 |
| ERV, l | 1.68 | ± | 0.38 | 1.74 | ± | 0.49 | 0.63 |
| ERV, % FVC | 35 | ± | 6 | 35 | ± | 6 | 0.92 |
| IRV, l | 4.10 | ± | 0.80 | 4.37 | ± | 0.88 | 0.13 |
| IRV, % FVC | 85 | ± | 8 | 89 | ± | 4 | 0.18 |
| V̇_E, CAP_, l⋅min^-1^ | 141 | ± | 36 | 151 | ± | 42 | 0.41 |
| V̇_E_/V̇_E, CAP_, % | 83 | ± | 12 | 80 | ± | 17 | 0.55 |
| Mean expired flow, l⋅s^-1^ | 4.07 | ± | 0.83 | 4.13 | ± | 0.96 | 0.68 |
|  |  |  |  |  |  |  |  |
| *EFL* |  |  |  |  |  |  |  |
| EFL, # | 9 | | | 7 | | | 0.77 |
| EFL, %V_T_ | 40 | ± | 24 | 43 | ± | 19 | 0.78 |

*Abbreviations:* EFL, expiratory flow limitation; ERV, expiratory reserve volume; F_B_, breathing frequency; FVC, forced vital capacity; HR, heart rate; IC, inspiratory capacity IRV, inspiratory reserve volume; RER, respiratory exchange ratio; V̇CO_2,_ carbon dioxide output; V̇_E_, minute ventilation; V̇_E,CAP_, ventilatory capacity; V̇_E_/V̇_E,CAP,_ fractional utilization of available ventilatory capacity; V̇O_2_, oxygen uptake; V_T_, tidal volume.

**Table S3**. Repeatability of metabolic and ventilatory parameters in the participants who experienced EFL during at least 1 of the 2 visits.

|  | CV, % | ICC | 95 % CI | *p* | | Repeatability  Classification |
| --- | --- | --- | --- | --- | --- | --- |
| *MEFV parameters* |  |  |  |  |  | |
| FVC, l | 2.73 | 0.99 | 0.97-1.00 | <0.001 | | *Excellent* |
| SR | 6.23 | 0.84 | 0.54-0.95 | <0.001 | | *Good* |
| FEF_25-75_, l⋅s^-1^ | 6.39 | 0.87 | 0.62-0.96 | <0.001 | | *Good* |
|  |  |  |  |  | |  |
| *Peak exercise* |  |  |  |  | |  |
| V̇O_2_, l⋅min^-1^ | 7.67 | 0.79 | 0.44-0.93 | <0.001 | | *Good* |
| V̇CO_2_, l⋅min^-1^ | 6.82 | 0.81 | 0.48-0.94 | <0.001 | | *Good* |
| V_T_, l | 4.38 | 0.78 | 0.41-0.93 | <0.001 | | *Good* |
| F_B_, breaths⋅min^-1^ | 6.45 | 0.56 | 0.03-0.85 | 0.020 | | *Moderate* |
| IC, l | 2.91 | 0.91 | 0.73-0.97 | <0.001 | | *Excellent* |
| ERV, l | 7.87 | 0.57 | 0.05-0.85 | 0.017 | | *Moderate* |
| IRV, l | 4.15 | 0.74 | 0.34-0.92 | 0.001 | | *Moderate* |
| Mean expired flow, l⋅s^-1^ | 4.23 | 0.87 | 0.62-0.96 | <0.001 | | *Good* |
| V̇_E_, l⋅min^-1^ | 5.03 | 0.82 | 0.5-0.94 | <0.001 | | *Good* |
| V̇_E, CAP_, l⋅min^-1^ | 9.11 | 0.46 | -0.11-0.8 | 0.052 | | *Poor* |
| V̇_E_/V̇_E, CAP_, % | 8.60 | 0.09 | -0.47-0.61 | 0.381 | | *Poor* |
|  |  |  |  |  | |  |
| *Matched V̇_E_* |  |  |  |  | |  |
| V̇O_2_, l⋅min^-1^ | 5.89 | 0.78 | 0.42-0.93 | <0.001 | | *Good* |
| V̇CO_2_, l⋅min^-1^ | 5.15 | 0.83 | 0.54-0.95 | <0.001 | | *Good* |
| V_T_, l | 3.01 | 0.91 | 0.73-0.97 | <0.001 | | *Excellent* |
| F_B_, breaths⋅min^-1^ | 3.89 | 0.91 | 0.73-0.97 | <0.001 | | *Excellent* |
| IC, l | 4.12 | 0.89 | 0.66-0.97 | <0.001 | | *Good* |
| ERV, l | 8.89 | 0.70 | 0.26-0.90 | 0.002 | | *Moderate* |
| IRV, l | 3.39 | 0.92 | 0.77-0.98 | <0.001 | | *Excellent* |
| V_T_/T_E_, l⋅s^-1^ | 4.18 | 0.91 | 0.72-0.97 | <0.001 | | *Excellent* |
| V̇_E_, l⋅min^-1^ | 1.65 | 0.99 | 0.98-1.00 | <0.001 | | *Excellent* |
| V̇_E, CAP_, l⋅min^-1^ | 7.32 | 0.78 | 0.41-0.93 | <0.001 | | *Good* |
| V̇_E_/V̇_E, CAP_, % | 7.43 | 0.82 | 0.51-0.94 | <0.001 | | *Good* |

*Abbreviations:* EFL, expiratory flow limitation; ERV, expiratory reserve volume; F_B_, breathing frequency; IC, inspiratory capacity IRV, inspiratory reserve volume; V̇_E_, minute ventilation; V̇_E,CAP_, ventilatory capacity; V̇_E_/V̇_E,CAP,_ fractional utilization of available ventilatory capacity; V_T_, tidal volume; V_T_/T_E_, mean expiratory flow.
